# Office Visitors

**Published:** 1907-03

**Authors:** 


### OFFICE VISITORS.

The door-bell, by the vigor of its ring, announced that the ringer was awfully in earnest. It is curious, yet true, that when a dentist is used to hearing his door-bell ring he can tell almost without error the frame of mind of the ringer. The office girl is also affected by it and answers either precipitately or at leisure as indicated. In the present instance the door was opened in a hurry and in walked a lady and little girl, the latter in tears and sobbing. "Hush right up now! I'm going to have something done to that tooth now or have it out. I've stood it long enough and am not to be kept awake at night any longer. I've tried to get you to come to the dentist this long while to have it pulled and you never would." "Oh, but mamma he will nearly kill me, I don't want it pulled" was plaintively wailed. "Nonsense, that is all owing to that bad Jennie Smart scaring you with her tales about the dentist. Come along now to the Doctor." Then the poor freckle faced, auburn haired little one was forcibly pulled into our operating room and amid the screams of the frightened child we were told to "put her in the chair, Doctor, and no matter if she does scream, pull it right out." Instead, we approach the child and gently taking and caressing a little hand and smoothing the red curls back from her wet face, we told her we did not desire to hurt her and maybe mamma was mistaken and that we expected we could put a little medicine in the tooth better than papa or mamma as we had done the day before for another little girl. We told her we would not put her in the big chair unless she wanted us to; but that it was a great old "elevator chair" and we gave all our little friends rides in it. We persuaded her to just let us look at the tooth and told her mother that it was a permanent tooth and entered into an argument in which we impressed the child that it was the mother who wanted the child to suffer and that we were her champion and friend, and if the mother would just let us we would stop the pain and not pull the tooth. The argument prevailed and bonny-red-hair got into the "elevator chair" and took several rides from the "first floor" to the

"top floor" and back. Particles of "breakfast and supper" were excavated to her astonishment and the water syringe was made to act as a "little garden fountain" and she was allowed "to run it". The dental engine was worked and the bur explained, how its "little teeth" which she felt on her finger "bit" the end of a tooth-brush handle. When we very carefully used it on her tooth and asked if it hurt she replied "it didn't hurt but it jiggled me". We so interested her that we were able to insert a devitalizing medicament and in the end got her in such a good humor by hoping her curls would not set the curtains afire" and that "her papa had a dollar to cover each freckle" that when told she must come back in a day or two she asked "wen mus I tum?" She did "tum back" and has been doing so for years now, to her "dear old Doctor".

